# Synthesis and Superficial Modification “In Situ” of Copper Selenide (Cu_2-x_ Se) Nanoparticles and Their Antibacterial Activity

**DOI:** 10.3390/nano14131151

**Published:** 2024-07-04

**Authors:** José Manuel Mata-Padilla, José Ángel Ledón-Smith, Marissa Pérez-Alvarez, Gregorio Cadenas-Pliego, Enrique Díaz Barriga-Castro, Odilia Pérez-Camacho, Christian Javier Cabello-Alvarado, Rodolfo Silva

**Affiliations:** 1 Centro de Investigación en Química Aplicada, Blvd. Enrique Reyna 140, Saltillo 25294, Coahuila, Mexico; jose.mata@ciqa.edu.mx (J.M.M.-P.); jangel.ledon.m20@ciqa.edu.mx (J.Á.L.-S.); enrique.diazbarriga@ciqa.edu.mx (E.D.B.-C.); odilia.perez@ciqa.edu.mx (O.P.-C.); christian.cabello@ciqa.edu.mx (C.J.C.-A.); 2 CONAHCYT-Centro de Investigación en Química Aplicada, Blvd. Enrique Reyna 140, Saltillo 25294, Coahuila, Mexico; 3 Universidad Nacional Autónoma de México, Instituto de Ingeniería UNAM, Ciudad Universitaria, México City 04510, Mexico; rsilvac@iingen.unam.mx

**Keywords:** copper selenide nanoparticles, gum arabic, antimicrobial susceptibility, chalcogenides, *Staphylococcus aureus*, *Escherichia coli*, *Candida albicans*

## Abstract

Copper selenide nanoparticles (Cu_2-x_ Se NPs) have received a lot of attention in recent decades due to their interesting properties and potential applications in various areas such as electronics, health, solar cells, etc. In this study, details of the synthesis and characterization of copper selenide nanoparticles modified with gum arabic (GA) are reported. Also, through transmission electronic microscopy (TEM) analysis, the transformation of the morphology and particle size of copper selenide nanoparticles in aqueous solution was studied. In addition, we present an antimicrobial study with different microorganisms such as *Staphylococcus aureus* (*S. aureus*), *Escherichia coli* (*E. coli*) and *Candida albiacans* (*C. albicans*). Copper selenide nanoparticles were characterized by X-ray diffraction (XRD), Fourier-transform infrared spectroscopy (FTIR), thermogravimetric analysis (TGA), differential scanning calorimetry analysis (DSC) and TEM. XRD confirmed the crystal-line structure of the nanoparticles such as cubic berzelanite with a particle size of 6 nm ± 0.5. FTIR and TGA corroborated the surface modification of copper selenide nanoparticles with gum arabic, and DSC suggested a change in the structural phase from cubic to hexagonal. TEM analysis demonstrated that the surface modification of the Cu_2-x_ Se NPs stabilized the nanostructure of the particles, preventing changes in the morphology and particle size. The antimicrobial susceptibility analysis of copper selenide nanoparticles indicated that they have the ability to inhibit the microbial growth of *Staphylococcus aureus*, *Escherichia coli* and *Candida albicans*.

## 1. Introduction

Due to their exceptional properties, copper nanoparticles have a wide spectrum of applications in several areas such as health, electronics, agriculture, etc., and the importance of copper nanoparticles compared to other metal nanoparticles is due to their high antimicrobial activity (AA) and conductivity [1,2,3]. Copper is one of the metallic elements that are essential to human health, being a constituent of hair and the elastic tissue contained in the skin, bone and other body organs [4]. In addition, a number of recent studies have explored the potential benefit of copper as a biocide [1,2,3]. The attractiveness of copper nanoparticles is mainly due to their higher abundance and lower cost compared to other elements like gold and silver [5], and a disadvantage is that they are easily oxidized, so they should be used alongside stabilizing agents to avoid this [6]. 

On the other hand, selenium nanoparticles have attracted a lot of attention in recent years due to their biocompatibility, bioavailability and low toxicity, since selenium is an essential micronutrient for the health of humans, microorganisms and animals. Due to their high bioactivity and excellent antimicrobial properties, selenium nanoparticles are mostly used in various biomedical applications [7]. In nature, selenium can be found in both crystalline and amorphous polymorphic structures. The crystalline forms of selenium are monoclinic and trigonal. Monoclinic selenium (m-Se) is red in color and contains rings of Se_8_. Based on different types of packing, it exists in three allotropic forms (α, β and γ). Trigonal selenium (t-Se) is black in color and is the most stable crystalline form at room temperature. Non-crystalline forms of selenium include red amorphous (a-Se), black amorphous, and vitreous selenium [8,9].

Metal chalcogenides have received enormous attention in the past few decades from researchers discovering their industrial applications. These are compounds that consist of at least one chalcogenide anion and at least one electronegative metal element. All of the elements in the VIA group of the periodic table are called chalcogens. Sulfides, selenides and tellurides are the anions more commonly referred to by the term metal chalcogenide. In particular, transition metals with empty orbitals can lead to the formation of nonstoichiometric compounds such as In_3_Se, Cu_1.8_Se, Cu_1.97_S, CuSe, Cu_2_S, etc. [10]. Copper-deficient copper selenide Cu_2-x_Se is a p-type semiconductor with direct and indirect bandgap energies in the ranges of 2.1–2.3 eV and 1.2–1.4 eV, respectively [11,12,13,14], and due to its excellent properties, it has many applications in biosensors [11], photothermal therapy [12], solar cells [13], electrodes [14], photoacoustic (PA) imaging [15], etc. One of the main properties of copper selenide nanoparticles is their antimicrobial activity, which has been studied by different research groups for applications in the medical area or where this type of property is required [16,17]. 

The surface functionalization of nanoparticles (NPs), during their synthesis, generally avoids agglomeration, prevents their growth and the formation of more active sites, decreases their toxicity and favors their dispersion in different media such as polymers, solvents, and paints, among others [18,19]. A high area/volume ratio leads to increased antimicrobial properties, and reports suggest that the close proximity between NPs and the bacterial cell membrane is a critical factor that controls the efficiency of NPs in bacterial inactivation. The NPs–bacterium interaction can be affected by aggregation and heteroaggegation related to the surface functionalization of NPs [20,21,22]. Generally, antimicrobial activity (AA) improves as the concentration of NPs increases, but some reports indicate that increased concentrations may lead to the aggregation of particles and bacterial cells that inhibit nontoxicity [23]. On the other hand, it is important to note that the AA of the metal nanoparticles is different toward Gram-positive and Gram-negative bacteria, and the AA depends on the interaction between the bacterial cell wall and the nanoparticle; different AA results for the same nanoparticle have been reported [24]. 

Recent applications of copper selenide compounds include the electrocatalytic conversion of CO_2_, where value-added chemicals such as hydrocarbons and alcohols are produced which can potentially reduce dependence on fossil fuels and reduce air pollution. The electrocatalysts generally use high-cost noble metals; recently, copper selenide compounds have emerged as an alternative to replace noble metals because they have multiple oxidation states, electrical conductivity, catalytic activity, and their cost is lower compared to that of noble metals. Also, unsaturated selenium atoms can increase the number of active sites [18,19].

There is a wide range of copper selenide structures such as CuSe, Cu_2_Se, Cu_2-x_Se, CuSe_2_, Cu_3_Se_2_, Cu_5_Se_4_, etc. [25,26,27], and also there are several crystallographic forms such as cubic, hexagonal, tetragonal, monoclinic and orthorhombic [25,28,29,30]. Copper selenides present a complicated characterization, because they have crystalline structures of variable composition (Cu_2-x_Se, where 0 ≤ x ≤ 1), and some products are unstable such as cuprous selenide (Cu_2_Se), which is easily oxidized to yield different stoichiometric compounds [31].

The synthesis of copper selenide nanostructures (nanowires, nanospheres, nanoplates, nanotubes, etc.) has been reported by a variety of methods such as pyrolysis, ultra-sonic, hydrothermal co-reduction, sol–gel, solvothermal, precipitation chemical, hot injection method, and electrodeposition [11,32,33,34,35]. As reported by Gurin et al., copper and copper selenide nanoparticles have been synthetized by the sol–gel method, where copper nitrate and selenium vapor were used as precursors [32]. Copper selenide nanoparticles were near-spherical and formed aggregated with a size up to 200–300 nm. A variation in the stoichiometry of Cu_2-x_Se from 1.75 a 2.0 exists, as determined by XRD. In other studio, the copper selenide nanoparticles were synthetized by hydrothermal co-reduction, they used CuSO_4_·5H_2_O and SeO_2_ as precursors of copper and selenium, respectively, and hydrazine as a reducing agent. They obtained Cu_2_Se nanoparticles of hexagonal flake shape with a side length of 1.3–2.0 µm [33]. Another investigation used bovine serum albumin (BSA) to obtain snake-like CuSe nanostructures by solution-phase chemical reaction. Cu(NO_3_)_2_ and selenium powder were used as precursors, Na_2_SO_3_ was used as a reducing agent, and BSA was used as the reaction medium and coating of the nanosnake; the ones presented in the range of 130.0 nm in length and 12.0 nm in width. The nanosnakes were crystalline and could be indexed to berzelianite Cu_2-x_Se. [36]. Also, the copper selenide nanoparticles have been synthetized by an ultrasonic chemical method, where Cu(CH_3_COO)_2_·H_2_O and selenium powder were precursors and hydrazine was used as a reducing agent. CuSe and Cu_2_Se nanoparticles were obtained with hexagonal and cubic crystalline structures, respectively; both nanoparticles presented different shapes, flake-shaped CuSe and earth-worm-like Cu_2_Se [34]. A method reported for synthetizing copper selenide nanoparticles is the chemical wet reduction, as described by Patidar et al. [37], where the high quality of the Cu_3_Se_2_ phase was synthetized through the solution-phase chemical reaction between copper and selenium. Precursors were copper and selenium powders, and hydrazine was used as a reducing agent. Cu_3_Se_2_ nanoparticles with a size of 25.0 nm were spherical in shape, having a tetragonal crystalline structure. Mbewana-Ntshanka et al. reported the synthesis of copper selenide, copper sulfide and copper oxide nanoparticles via the hot-injection method, where oleylamine was used as a stabilizer agent and CuCl_2_, selenium and sulfur powders were used as precursors [9]. They obtained spherical nanoparticles in the ranges of 1.0–27.0 nm for CuSe, 1.0–18.0 nm for CuS and 0.1–8.0 nm for CuO. Singh et al. [14] reported another method for producing copper selenide nanoparticles: the solvothermal method, which involves solvents and thermal treatment. SeO_2_ and CuO were precursors of copper and selenium, and polyvinylpolymide (PVP) was used as a stabilizing agent. They synthetized a series of copper selenides with different stoichiometric and structural phases like β-Cu_1.3_Se, α-Cu_1.8_Se, α-Cu_2.5_Se, α-Cu_2.7_Se, and α-Cu_2_Se, and used water/EG/PVP as a reaction media. They suggested that the degree of crystallinity and crystalline size tend to increase with reaction temperature. It is well understood that pure selenium nanoparticles are not stable in aqueous medium because their morphology and size change depending on temperature and storage time; synthesis in the presence of macromolecules with polar groups stabilizes the nanostructure of the particles, avoiding morphological changes; this same behavior was observed in the copper selenide synthesis with bovine serum albumin as a foaming agent [36]. 

In most of the research developed for synthetizing copper selenide nanoparticles, their stoichiometric shape and crystalline structure largely depend on the synthesis method, reaction time, reaction temperature, Cu/Se ratio, and reducing and stabilizing agents, among others. This work presents the “in situ” synthesis and surface modification of copper selenide nanoparticles by chemical reduction. The method is easy, practical, and straightforward. It could be considered environmentally friendly since it used gum arabic as a stabilizing agent and water as a reaction medium. Furthermore, this method and reaction conditions allow only one type of crystalline structure, like berzelianite. Antimicrobial susceptibility to *Staphylococcus aureus*, *Escherichia coli* and *Candida albicans* was also studied.

## 2. Materials and Methods

### 2.1. Materials

The synthesis and surface modification of Cu_2-x_Se nanoparticles was carried out using a naturally occurring polysaccharide such as gum arabic (GA) ≥ 97.0%, copper (II) sulfate pentahydrate (CuSO_4_·5H_2_O) ≥ 98.0%, hydrazine hydrate (N_2_H_4_·H_2_O, reagent grade, 50–60%), and selenous acid (H_2_SeO_3_, 97.0%). All reagents were supplied by Sigma-Aldrich (St. Louis, MO, USA). The microbial strains *Staphylococcus aureus* (Gram-positive), *Escherichia coli* (Gram-negative) and *Candida albicans* (fungi) were provided by American Type Culture Collection (ATCC), (Manassas, VA, USA).

### 2.2. Synthesis of Copper Selenide (Cu_2-x_ Se) Nanoparticles

**Scheme 1:**
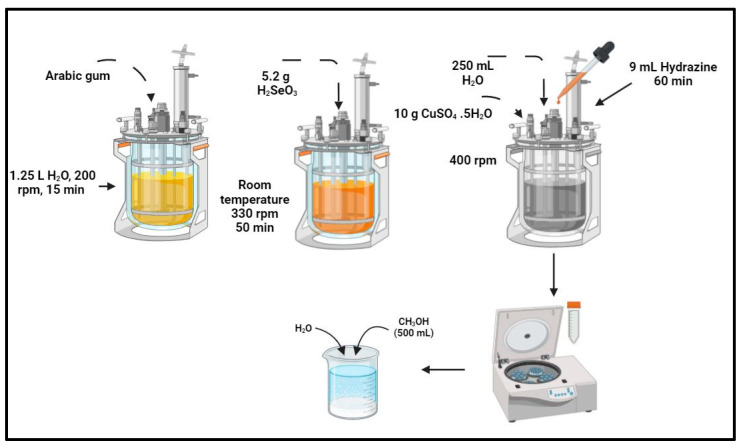
Synthesis of the Cu_2-x_ Se NPs superficially modified with gum arabic.

The chemical synthesis of CuSe nanoparticles was carried out in our working group and previously reported [38]; Scheme 1 shows the chemical synthesis of copper selenide nanoparticles.

### 2.3. Antimicrobial Analysis

The antimicrobial susceptibility of synthesized copper selenide nanoparticles was analyzed using the agar well diffusion method [39]. The strains of microorganisms employed were *Staphylococcus aureus* (Gram positive), *Escherichia coli* (Gram negative) and *Candida albicans* (fungi). The microorganism colonies were put in a saline solution until a measure in UV-Vis of 0.1 of absorbance in a 600 nm wavelength, which is equivalent to 0.5 of bacteriological suspension turbidity in the McFarland standard (1 × 10^8^ colony-forming units, CFU/mL) [40]. First, BD Bioxon agar was prepared and sterilized, which was placed on plates, and after the gelation, the surface of the plate was completely rubbed with continuous rotation to create a uniform layer of bacteria (CL experiment), using a sterile cotton swab.

For each microorganism, five concentrations of NPs were studied (1.0%, 2.0%, 3.0%, 4.0% and 8.0% by weight). Four holes of 6 mm diameter were drilled in each of the agar plates; then, 20 µL of an aqueous solution containing copper selenide nanoparticles at different concentrations was deposited in each hole. The plates were labeled and incubated at 37 °C for 24 h. All antimicrobial experiments were duplicated with four replicates per plate to ensure reproducibility and results.

### 2.4. Characterization Techniques

X-ray diffraction (XRD): For this assay, a Siemens D-5000 diffractometer with a scanning interval in the 2θ scale from 20° to 80° and a scan speed of 0.02% was used (Siemens, Berlin, Germany). Copper K_α_ radiation was employed with a wavelength of 1.54 Å. Values of 25 mA and 35 kV were used for intensity and voltage, respectively. 

Thermogravimetric analysis (TGA): The TGA 5500 Discovery analyzer (TA instruments Inc., New Castle, PA, USA) was used for this characterization under carefully controlled conditions. The operating conditions included a heating rate of 10 °C/min and an air atmosphere with a gas flow of 50 mL/min. The samples were run from 30 to 600 °C in an N_2_ atmosphere; once 600 °C was reached, the N_2_ atmosphere was switched to O_2_ for the more efficient combustion of organic components, ensuring reliable and reproducible results.

Differential scanning calorimetry analysis (DSC): The DSC 2500 Discovery DSC series (TA instruments Inc., New Castle, PA, USA) was used for this test. The operating conditions included a heating rate of 10 °C/min in a temperature range from 40 to 160 °C. A second heating cycle was performed to eliminate the sample’s thermal and mechanical history, ensuring a clean and accurate analysis

Fourier-transform infrared spectroscopy (FTIR): A spectrometer Magna Nicolet 550 (GMI trade) was used to obtain the FTIR spectra for this characterization (GMI, Minneapolis, Minnesota, USA). Copper selenide nanoparticles were blended with moisture-free potassium bromide (KBr), and a thin disk was prepared with this mixture. The spectral range analyzed was from 400 to 4000 cm^−1^. This analysis was used to identify the presence of the organic coatings on the nanoparticles surface.

Transmission electronic microscopy (TEM): For this test, copper selenide nanoparticles were scattered in distilled water using an ultrasonic device, and subsequently, some drops were put in carbon-coated nickel grilles, and afterwards, they were left to dry in a free environment powder at room temperature. The equipment used was an FEI Titan high-resolution transmission electron microscope model 80–300 kV operating at 300 kV (FEI Company, Hillsboro, OR, USA).

## 3. Results and Discussion 

### 3.1. X-ray Diffraction Analysis of Copper Selenide (Cu_2-x_ Se) Nanoparticles

**Figure 1:**
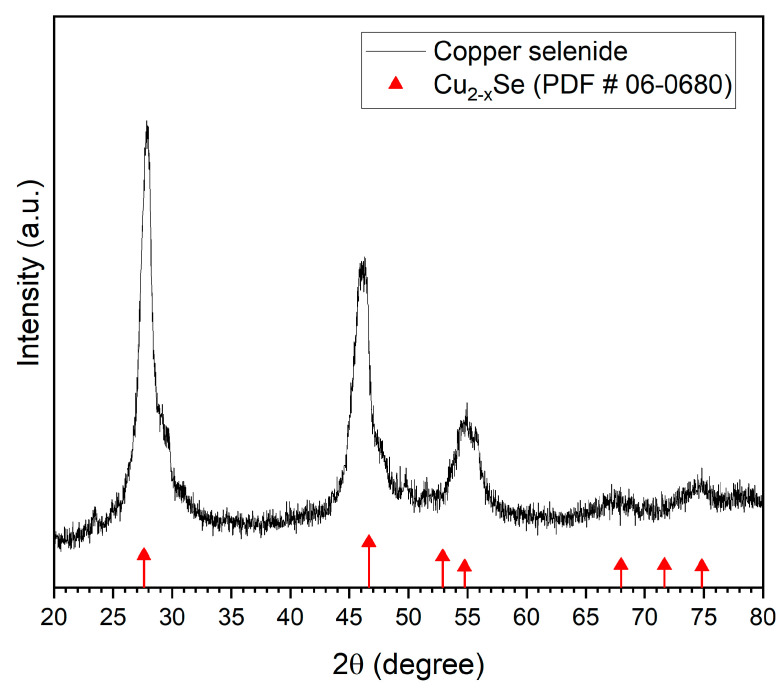
XRD patterns of copper selenide nanoparticles obtained and standard copper selenide (Cu_2-x_ Se) (PDF # 06-0680).

Figure 1 shows the X-ray diffractograms of the copper selenide nanoparticles synthetized and modified using gum arabic. The results show that the copper selenide synthetized is of a type called cubic berzelianite Cu_2-x_ Se (PDF #06-0680), which presents characteristic peaks at 27.7°, 45.9°, 54.7°, 67.9° and 74.6° on a 2 theta (2θ) scale, and its Miller planes are (111), (211), (311), (400) and (331), respectively [11,41]. The localization and intensity of XRD peaks (Figure 1) may differ from the literature reports. Similar observations where copper selenide was stabilized with several organic ligands were reported [20,21].

Surprisingly, there are no peaks characteristic of copper oxide (Cu_2_O or CuO species) or pure copper (Cu°), even though the reaction was carried out in an aqueous medium, which suggests that gum arabic is a good stabilizing agent of synthesized nanoparticles. Therefore, the oxidation resistance property of copper nanoparticles could be attributed to gum arabic and, consequently, the oxygen and moisture entry is prevented [42,43]. The presence of amorphous phase selenium is discarded, since this phase presents broad peaks of up to 10° between 2*θ* = 10–30° [44]. However, the crystalline phase of hexagonal selenium is completely discarded, since this phase presents the main peaks characteristic at 2θ values of 14.46°, 32.39°, 40.25°, 46.45°, 47.95°, 49.63°, 52.81° and 67.56° approximately, corresponding to the Miller planes (100), (101), (110), (102), (111), (201), (112) and (210), respectively (JCPDS file No. 06-0362) [45]. Also, the crystalline phase of trigonal Se is completely discarded, since this phase shows the peak characteristic at 2θ values of 23.0, 29.0, 41.0, 43.0, 45.0, 51.0, 55.0, 61.0 and 65.0°, corresponding to the crystalline planes (100), (101), (110), (102), (111), (201), (112), (103), and (210) according to (JCPDS file No. 651876) [46].

The average crystallite size of copper selenide nanoparticles was estimated from the corresponding X-ray diffraction peak employing the Debye–Scherrer’s formula [47].

D=κλ/(β cosθ)(1)

Here, κ is an empirical constant equal to 0.94, λ is the wavelength of the X-ray source (1.5405 Å), β is the full width at half maximum of the diffraction peak and θ is the angular position of the peak. The average crystallite size calculated by the Debye–Scherrer equation was 6.0 nm ± 0.5, the determination was made with the most intense peak located at 27.7 in the 2θ scale.

### 3.2. Fourier Transformed Infrared Spectroscopy (FTIR)

**Figure 2:**
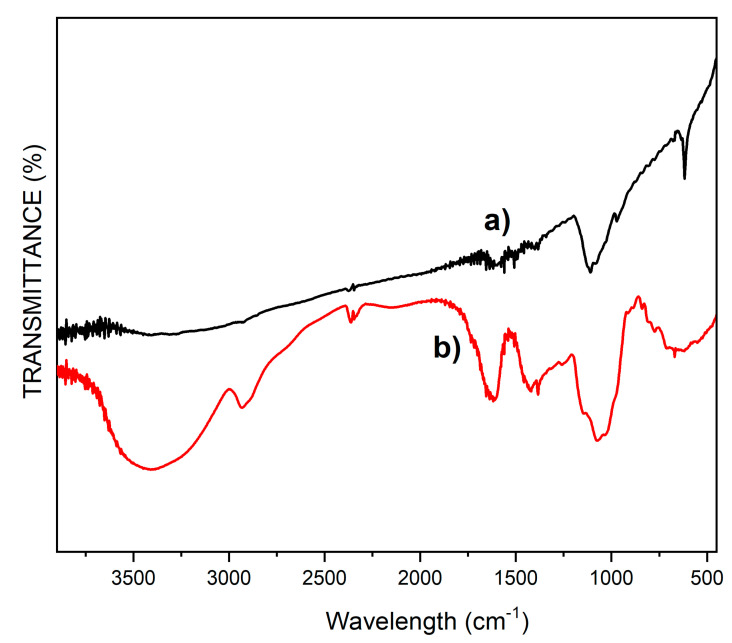
FTIR spectra of (a) gum arabic and (b) copper selenide nanoparticles modified.

Figure 2 shows the spectrum of copper selenide nanoparticles (Figure 2a) synthetized by biochemical reduction and gum arabic (Figure 2b), which was used as a stabilizing and modifying agent.

The gum arabic spectrum shows some characteristics signs localized between 1073.7 and 1420.2 cm^−1^ that are assigned to the stretching of the C-O bond and the bending of the OH^−^ group of the carboxyl group (COOH). The bands at 1609.6 and 1637 cm^−1^ correspond to the symmetrical and asymmetric stretching of the COO^−^ group and the stretching of the C=O bond, respectively. The signal at 2931.4 cm^−1^ is attributed to the vibration of the C-H bond (aliphatic groups). The absorption band shown at 2360 cm^−1^ is usually due to CO_2_ vibrations. The absorption band located at 3417.1 cm^−1^ corresponds to an O-H bond stretch [42,44].

On the other side, we can see the spectrum of copper selenide nanoparticles modified with GA (Figure 2b) that presented a decrease in the intensity of the signals at 1420, 1609 and 3417.1 cm^−1^, which was attributed to GA. The same situation is given with the absorption band localized at 2931 cm^−1^ belonging to C-H bonds. To be more specific, the rubber signals attributed to the bending of the OH of the COOH group and the stretching of the COO^−^ group were significantly reduced, and the signal of the COO^−^ group was displaced from 1609 to 1618 cm^−1^. Something similar occurred in the band at 3417.1 cm^−1^ attributed to the stretching of the OH group, which appears to flatten the zone. This behavior suggests and indicates the interaction of hydroxyl groups of gum arabic on the surface of copper selenide nanoparticles; this binding could be preventing the movement of these groups [42,43,44,48]. On the other hand, the presence of the band at 607 cm^−1^ is attributed to the bending vibration of the copper selenide bond [23,49]. 

### 3.3. Thermogravimetric Analysis (TGA)

**Figure 3:**
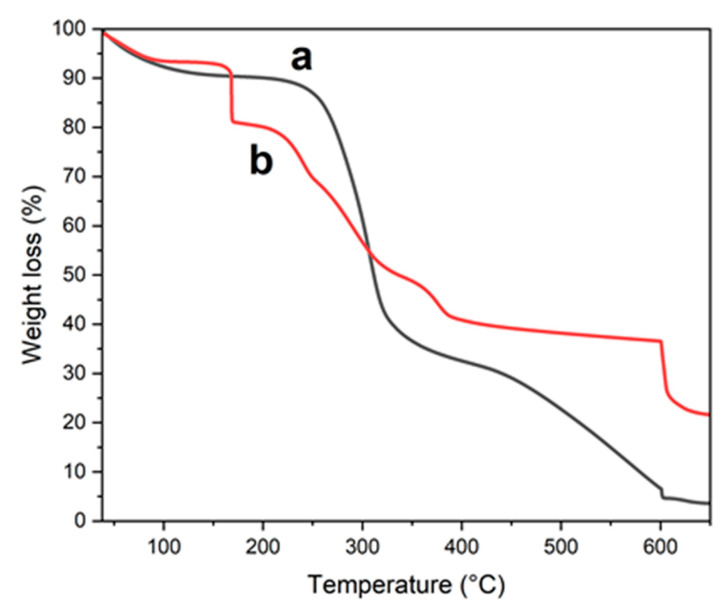
TGA thermograms of (a) gum arabic and (b) modified copper selenide nanoparticles with gum arabic.

The synthesized copper selenide nanoparticles were analyzed by TGA. Figure 3 shows the TGA thermograms of the synthetized copper–selenide/GA nanoparticles and gum arabic.

As seen in Figure 3a, the decomposition of gum arabic presents three stages of mass loss from 30 to 750 °C. The decomposition of gum arabic occurs in the range of 170–600 °C, and the first weight loss of 9.63%, between 30 and 170 °C, is attributed to the elimination of water present on the surface and water attached to the structure [50,51,52]. The second weight loss of 58.21% occurred in the range of 170–405 °C, which can be associated with the denaturation of the gum polysaccharides due to the decomposition of carboxylate groups and the carboxylic acids present [53]. In the literature, the decomposition initiation temperatures of the first stage depend on the nature of the gum arabic used [50]. The third weight loss occurred in the range of 405–590 °C, which presents a loss of 24.0%. It is essential to mention that for the sample used in this investigation, the most significant decomposition of the gum arabic is achieved at these two last weight loss stages from 170 to 590 °C. After the third weight loss, there are two additional weight losses of 3.57% and 1.04% by weight, which correspond to the losses found between 590 and 610 °C and between 610 and 665 °C, respectively. The percentage residual was 3.55%, which is similar to studies with structures different from those modified and without modified gum arabic [54,55]. The results of DTG indicate that the maximum degradation temperatures (T_máx_) found at 307 °C, 550 °C, 600 °C and 624.7 °C were according to the values reported in the literature [32,53]. Also, in Figure 3b the thermogram of modified copper selenide nanoparticles can be observed, which exhibits six decomposition stages, while three decomposition stages were observed for GA. The decomposition stages of copper selenide nanoparticles could be attributed to the gradual volatilization of selenium, leading to the formation of copper-enriched specimens during the analysis process due to thermal instability [35]. The copper selenide nanoparticles thermogram (Figure 3b) shows the first weight loss at 54 °C is attributed to the bound surface water molecules present in the sample, while the second weight loss in the 110–190 °C range can be attributed to the vaporization and removal of occlusion water in the modified Cu_2-x_Se nanoparticles, which indicate the characteristic of moisture sorption caused by radical hydroxyl groups [56] or also to monoclinic selenium decomposition, since its initial decomposition temperature is 180 °C [57]. It is well known that the main component of gum arabic is a highly branched polysaccharide that is structurally constituted by β-(1,3) galactose bound with arabinose and rhamnose with glucuronic acid endings. Therefore, the other decomposition stages are most likely due to the decomposition of these compounds. For example, galactose is reported to have a decomposition temperature of 282 °C, and thermal analysis studies corroborate this [58]. However, it could also be due to the decomposition of selenium with a trigonal structure, since its initial decomposition temperature is 200 °C [57]; for amorphous selenium, its initial decomposition temperature is 70 °C [59,60]. Garba et al. reported that the complete decomposition of CuSe is 447.08 °C for samples coated with tartaric acid and oxygen as the atmosphere [49]. Below this temperature, it is considered that most of the selenium was volatilized; the application of different binders on the surface of particles can change this decomposition temperature.

### 3.4. Differential Scanning Calorimetry (DSC)

**Figure 4:**
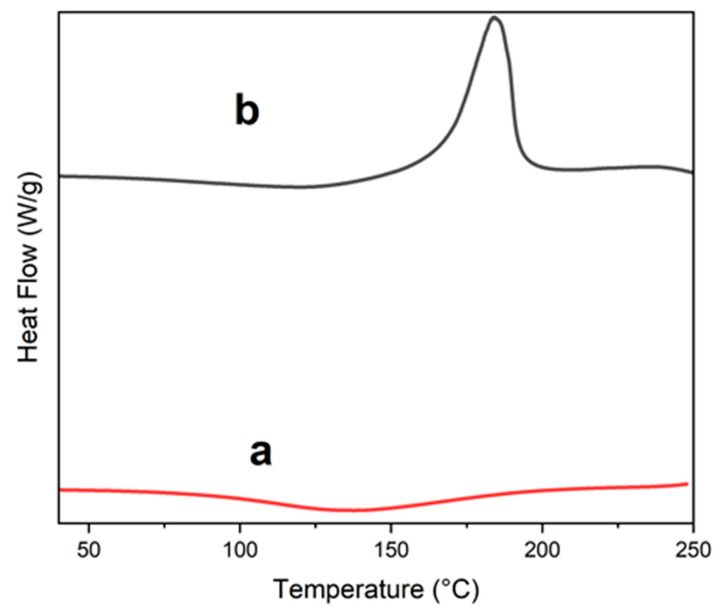
DSC thermograms of (a) gum arabic and (b) surface-modified copper selenide nanoparticles.

The samples were analyzed by DSC; Figure 4 shows the gum arabic and surface-modified copper selenide nanoparticles thermograms. 

The gum arabic thermogram (Figure 4a) exhibits an endothermic peak at 138 °C, which begins at 70 °C and ends at 210 °C; this can be attributed to water encapsulated into the crystalline structure of the material, superficial water, and water absorbed physically preceding the hydrophilic character [60]. Also, this transition can be caused by the partial decomposition of functional groups of gum arabic [52]. In Figure 4b, the endothermic peak located at 273 °C corresponds to the melting point of elemental selenium [61], suggesting the presence of this element, which was not detected by XRD. Its presence can also be explained by the decomposition of the compound Cu_2-x_Se in CuSe and elemental selenium, which can occur at temperatures greater than 250 °C [61]. By increasing the temperature, the endothermic process by 338 °C confirms the decomposition of CuSe into β-Cu_2-x_Se and Se [62].

### 3.5. Transmission Electronic Microscopy (TEM)

**Figure 5:**
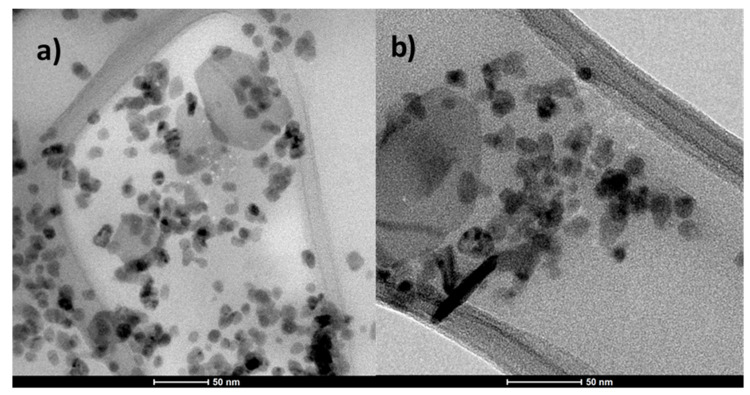
Micrographs of copper selenide nanoparticles modified with gum arabic. (**a**) Nanoparticles isolated after one day of synthesis, (**b**) Nanoparticles isolated after two days of synthesis.

Selenium nanoparticles and derivatives of coper selenide are not readily established in an aqueous medium: their stability is contingent upon various factors, including the temperature and type of stabilizing agent, among others [25,26,27,28,29,30,31]. The Cu_2-x_Se/GA obtained were meticulously analyzed using transmission electronic microscopy (TEM). Nanoparticles of copper selenide were isolated one and two days after the conclusion of the reaction and after TEM analysis (Figure 5).

Analysis of the TEM images revealed a fascinating phenomenon: the changes in the size and morphology of copper selenide nanoparticles were corroborated. TEM micrographs of the Cu_2-x_Se/GA nanoparticles, separated after one day, showed hemispherical particles (Figure 5a) with an average diameter mainly of 7.0 nm. The nanoparticles tend to coalesce or join together, forming elongated nanosnake-like particles, similar to those previously reported [36]. Rod-sharped nanoparticles with an average length of 50.0 nm and width of 5.0 nm were also observed (Figure 5b).

**Figure 6:**
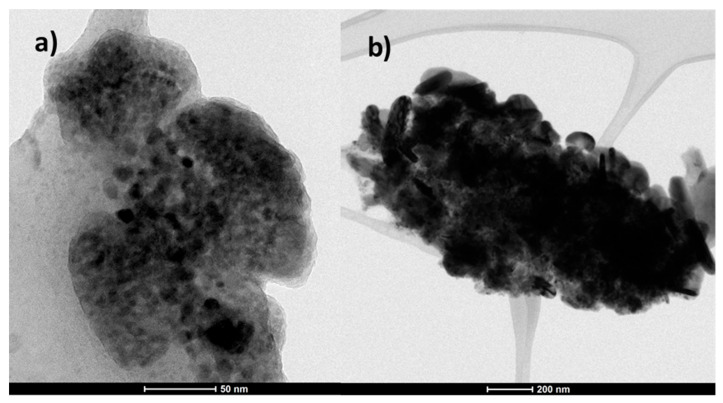
TEM micrographs of copper selenide nanoparticles modified with gum arabic. (**a**) Nanoparticles isolated after one day of synthesis, (**b**) Nanoparticles isolated after two days of synthesis.

TEM micrographs (Figure 6) of the Cu_2-x_Se/GA nanoparticles, separated after two days, presented aggregates of irregular shape with an approximate length of 200–800 nm. These agglomerates are made up of hemispheric subparticles with a size of 6.0–11.0 nm (Figure 6a), and there are other agglomerates with hemispheric and elongated shapes that were larger than 200 nm in length (Figure 6b). This suggest that gum arabic may be responsible for the aggregates formation and also for preventing a complete morphologic transformation of the hemispheric nanoparticles.

**Figure 7:**
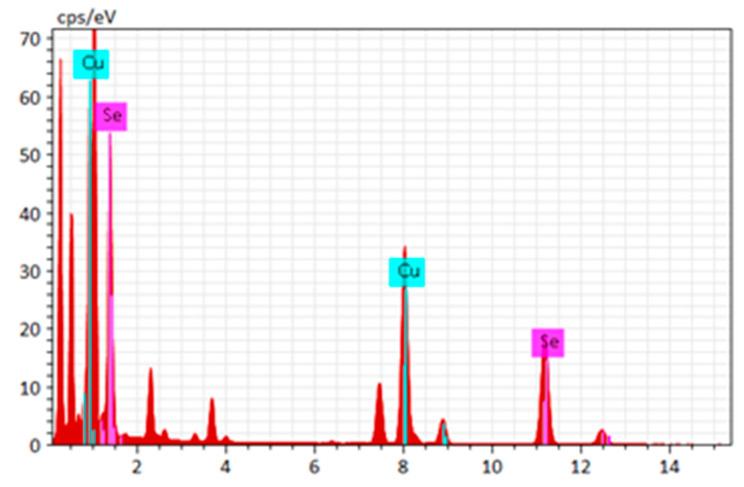
EDS-TEM analysis copper selenide nanoparticles.

To determine the composition profile of Cu_2-x_ Se/GA nanoparticles, the sample was analyzed using EDS-TEM (Figure 7), where the quantification yields of the composition profile of NPs can be observed. 

Quantification of the EDS spectrum yields the composition profile demonstrating the compositional structure of copper selenide; the elements of carbon, oxygen, copper and selenium can be observed. The signal localized at 7.5 keV corresponds to nickel, which belongs to the grid material used in the analysis. Other signals are localized at 2.25 and 3.75 keV without labels, and they belonged to sulfur and calcium, respectively. These elements are impurities that were probably taken during the preparation process or the sample handing. The Se/Cu ratio obtained by this technique was 1.24; this result is due to the theoretical relationship of the elements based on the amount of reagents used. 

### 3.6. Antimicrobial Sensitivity

**Figure 8:**
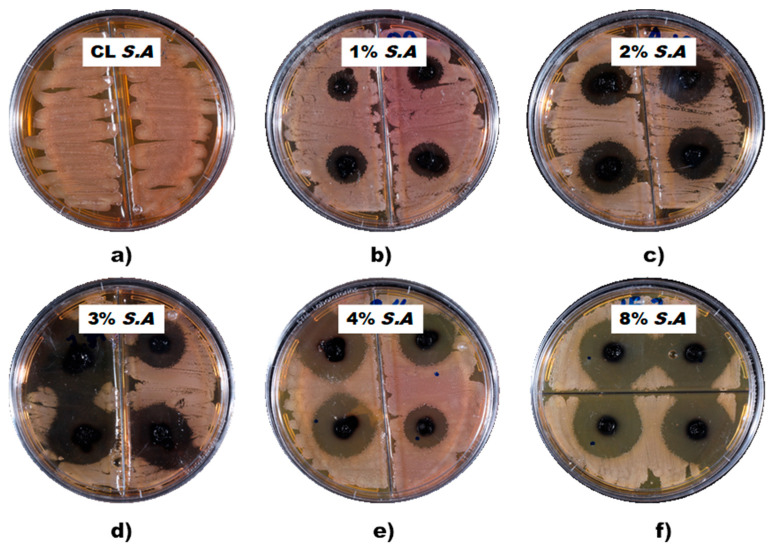
Antibacterial activity of copper selenide nanoparticles at different concentrations: (**b**) 1.0, (**c**) 2.0, (**d**) 3.0, (**e**) 4.0 and (**f**) 8.0% weight against *Staphylococcus aureus* (**a**).

**Figure 9:**
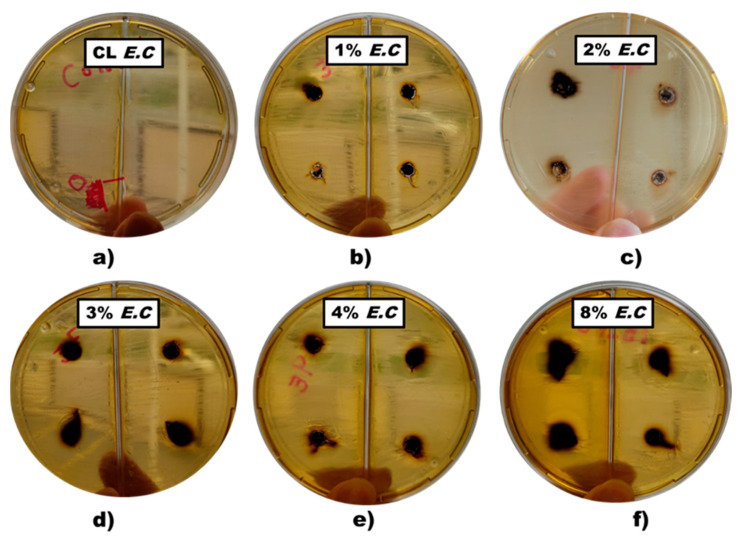
Antibacterial activity of copper selenide nanoparticles at different concentrations: (**b**) 1.0, (**c**) 2.0, (**d**) 3.0, (**e**) 4.0 and (**f**) 8.0% weight against *Escherichia coli* (**a**).

**Figure 10:**
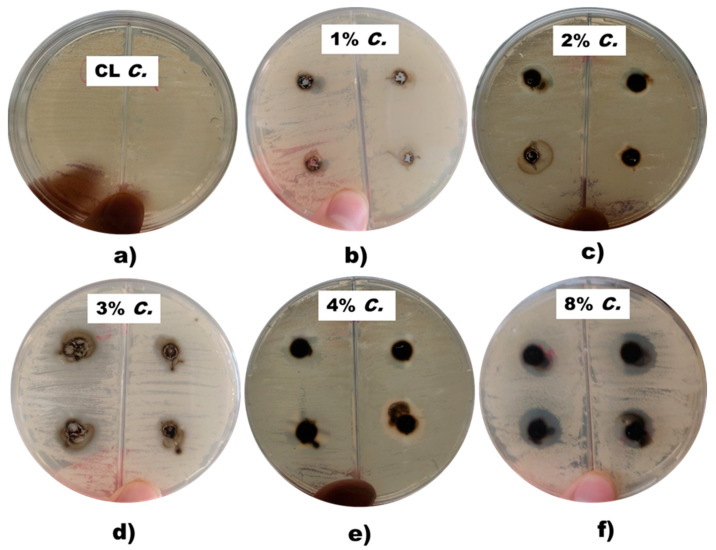
Antibacterial activity of copper selenide nanoparticles at different concentrations: (**b**) 1.0, (**c**) 2.0, (**d**) 3.0, (**e**) 4.0 and (**f**) 8.0% weight against *Candida albicans* (**a**).

The antimicrobial sensitivity of copper selenide nanoparticles was determined by the agar well diffusion method. Figure 8 shows the images obtained from the evaluation experiment with *Staphylococcus aureus* (Gram positive), Figure 9 shows the images from the evaluation experiment with *Escherichia coli* (Gram negative), and Figure 10 shows the images obtained from the evaluation experiment with *Candida albicans*. 

The effectiveness of the copper selenide nanoparticles in inhibiting microbial growth is evident in the increases in the size of the inhibition halo, as seen in all of the figures, with the increased concentration of the nanoparticles. This trend is particularly pronounced in the case of *S. aureus* bacteria. The solutions used for antimicrobial assessment were prepared using distilled water and weight percentages of 1.0, 2.0, 3.0, 4.0 and 8.0 of copper selenide nanoparticles. And these five solutions with different concentrations of nanoparticles were prepared for each strain (*E. coli*, *S. aureus*, *C. albicans*). CL is the key to identifying the control experiment (which does not have copper selenide nanoparticles), and within the figures, the images are labeled according to (a) which is the control, (b) the nanoparticles concentration of 1.0% weight, and successively until (f), which has the one with the highest concentration of nanoparticles (8.0%).

Figure 8 shows the bacterial growth inhibitory power of copper selenide nanoparticles against *S. aureus* bacteria, which is a Gram-positive specimen, by preprinting a 15 mm halo with only 1.0% nanoparticle weight. The inhibition present in the medium is attributed to the release of copper and selenium ions.

Despite using the same concentration of copper selenide nanoparticles (1.0% by weight), the behavior is different from that of *E. coli* bacteria (Figure 9), since the size of the inhibiting halo was reduced by almost half (8.21 mm). This is due to the unique properties of the outer layer of lipopolysaccharide of Gram-negative bacteria that prevents the adhesion of nanoparticles and exhibits high resistance to different biocides. The outer membrane, composed of a lipopolysaccharide complex that acts as endotoxins, hinders the passage of small hydrophilic molecules. It is important to note that endotoxins are structural components exclusively of Gram-negative bacteria [63].

Figure 9 shows how the bacterial inhibition halo increases in size as the concentration of nanoparticles increases, showing that 8.0% has an inhibition halo of approximately 17.175 mm. The copper nanoparticles exhibit lower growth inhibition with *E. coli* compared to *S. aureus.* This is probably due to the intrinsic resistance that could occur with Cu_2-x_Se nanoparticles, with the hydrophilic surface of its outer membrane that is rich in liposaccharides forming an impermeable barrier against toxic agents, avoiding the passage of molecules. 

Although the evaluation of copper selenide nanoparticles as bacterial growth inhibitors was performed under the same conditions against the bacterial strains *E. coli* and *S. aureus*, Gram negative and Gram positive, respectively, the power of copper selenide nanoparticles is higher toward *S. aureus*; these results are consistent with those reported, but there are still some controversies of the topic [24,63]. It is important to note that the chemical structure of the cell wall of bacteria plays an important role in giving a complete explanation of the bacterial growth inhibition of Gram positive and Gram negative, as this it is essential to carry out more specific studies to really know the mechanism of bacterial inhibition.

Figure 10 presents the images of the evaluation of copper selenide nanoparticles as inhibitors of fungal growth against *Candida albicans*. It can also be seen how the inhibitory halo increases in size as the concentration of nanoparticles increases (8.0% concentration correlated with about 21.275 mm. If this value is compared with the value obtained in *E. Coli*, it is much higher. This is probably due to the compounds in its cell wall, which are glycoproteins, chitin, and glucans. Mbewana-Ntshanka et al. [9] conclude that copper oxide nanoparticles have more antibacterial power against *E. coli* and *C. albicans* than copper chalcogens (CuSe and CuS). The average halo size values found in each of the antimicrobial evaluation experiments carried out are shown in Table 1 below. 

The purpose of obtaining superficially modified Cu_2-x_Se NPs with GA, in high-weight percentages, was to stabilize the nanostructure and avoid the change in morphology and particle size through synthesis conditions as well as improve the antimicrobial properties [3,18,19]. It is important to note that antimicrobial properties, due to their nanometric scale, can be lost by the large amount of GA on the surface of Cu_2-x_Se NPs and also by the high concentrations used during the antimicrobial evaluation [20,21,22,23]. Another aspect to take into account is that the results described in this section demonstrate that the antimicrobial evaluation was not affected by these situations. It is also of great relevance, and it is emphasized that to our knowledge, there are no reports on the bacterial growth inhibition study of Cu_2-x_Se nanoparticles with this stoichiometry.

## 4. Conclusions

In this investigation, it was possible to obtain a chalcogenide such as surface-modified copper selenide through an “in situ”, practical, easy, and simple methodology. The reaction conditions using lead for the formation of copper selenide nanoparticles with a cubic crystalline structure called berzelianite with a stoichiometric Cu_2-x_Se shape. Gum arabic is an excellent stabilizing agent and coating of copper selenide nanoparticles, and it is also environmentally friendly. 

The use of copper and gum arabic for obtaining copper selenide nanoparticles prevents the morphological change in selenium, since it is unstable and tends to change its morphology and size in an aqueous medium. The large amount of gum arabic covering the surface of the nanoparticles did not affect the antimicrobial activity, suggesting that the agglomeration of nanoparticles is low, as observed by TEM analyses. The Cu_2-x_Se nanoparticles showed inhibition zones in the microbial growth of all the strains of microorganisms tested (*E. coli, S. aureus and C. albicans*), which are more effective against strain *S. aureus*. In conclusion, we observed that the diameter of the inhibition zone increases as the concentration of nanoparticles increases. It is important to note that despite the high percentages of antimicrobial evaluation (1.0–8.0 wt %), there was no decrease in antimicrobial activity.

## Figures and Tables

**Table 1:** The results of measuring the diameter in mm of the inhibition halo and standard deviation.

Sample	Concentration (% wt.)	Microorganism	Average Inhibition Diameter (mm) and Standard Deviation
CL S.A.	0	*Staphylococcus aureus*	0
1.0% S.A.	1.0	*Staphylococcus aureus*	15.1 ± 1.2
2.0% S.A.	2.0	*Staphylococcus aureus*	21.1 ± 2.9
3.0% S.A.	3.0	*Staphylococcus aureus*	23.9 ± 3.3
4.0% S.A.	4.0	*Staphylococcus aureus*	24.0 ± 2.2
8% S.A.	8.0	*Staphylococcus aureus*	29.2 ± 1.8
CL C.	0	*Candida albicans*	0
1.0% C.	1.0	*Candida albicans*	8.1 ±1.8
2.0% C.	2.0	*Candida albicans*	15.2 ± 1.0
3.0% C.	3.0	*Candida albicans*	17.3 ± 1.0
4.0% C.	4.0	*Candida albicans*	18.3 ± 2.2
8.0% C.	8.0	*Candida albicans*	21.3 ± 1.2
CL E.C.	0	*Escherichia coli*	0
1.0% E.C.	1.0	*Escherichia coli*	8.2 ± 0.70
2.0% E.C.	2.0	*Escherichia coli*	11.9 ± 2.3
3.0% E.C.	3.0	*Escherichia coli*	13.8 ± 2.0
4.0% E.C.	4.0	*Escherichia coli*	15.6 ± 1.2
8.0% E.C.	8.0	*Escherichia coli*	17.2 ± 0.40

## Data Availability

Date are contained within the article.
